# Fatty Acid-Binding Protein 5 Modulates Brain Endocannabinoid Tone and Retrograde Signaling in the Striatum

**DOI:** 10.3389/fncel.2022.936939

**Published:** 2022-07-07

**Authors:** Mohammad Fauzan, Saida Oubraim, Mei Yu, Sherrye T. Glaser, Martin Kaczocha, Samir Haj-Dahmane

**Affiliations:** ^1^Department of Anesthesiology, Renaissance School of Medicine, Stony Brook University, Stony Brook, NY, United States; ^2^Department of Biochemistry and Cell Biology, Stony Brook University, Stony Brook, NY, United States; ^3^Department of Pharmacology and Toxicology, Jacobs School of Medicine and Biomedical Sciences, University at Buffalo, State University of New York, Buffalo, NY, United States; ^4^Department of Biological Sciences, Kingsborough Community College, Brooklyn, NY, United States; ^5^University at Buffalo Neuroscience Program, Jacobs School of Medicine and Biomedical Sciences, University at Buffalo, State University of New York, Buffalo, NY, United States

**Keywords:** endocannabinoid, FABP, fatty acid-binding protein, striatum, medium spiny neurons, anandamide, 2-arachidonoylglycerol, GABA synapses

## Abstract

The endocannabinoid (eCB) anandamide (AEA) and 2-arachidonoylglycerol (2-AG) are endogenous lipid neurotransmitters that regulate an array of physiological functions, including pain, stress homeostasis, and reward. Fatty acid-binding protein 5 (FABP5) is a key modulator of intracellular eCB transport and inactivation. Recent evidence suggests that FABP5 controls synaptic 2-AG signaling at excitatory synapses in the dorsal raphe nucleus. However, it is currently not known whether this function extends to other brain areas. To address this, we first profiled eCB levels across several brain areas in FABP5 knockout mice and wild-type controls and report that FABP5 deletion elevates AEA levels in the striatum, prefrontal cortex, midbrain, and thalamus, as well as midbrain 2-AG levels. The expression of eCB biosynthetic and catabolic enzymes was largely unaltered in these regions, although minor sex and region-specific changes in the expression of 2-AG catabolic enzymes were observed in female FABP5 KO mice. Robust FABP5 expression was observed in the striatum, a region where both AEA and 2-AG control synaptic transmission. Deletion of FABP5 impaired tonic 2-AG and AEA signaling at striatal GABA synapses of medium spiny neurons, and blunted phasic 2-AG mediated short-term synaptic plasticity without altering CB1R expression or function. Collectively, these results support the role of FABP5 as a key regulator of eCB signaling at excitatory and inhibitory synapses in the brain.

## Introduction

The endocannabinoids (eCBs) anandamide (AEA) and 2-arachidonoylglycerol (2-AG) are signaling lipids that activate cannabinoid type-1 (CB1R) and type-2 (CB2R) receptors in the central nervous system and peripheral tissues (Zou and Kumar, 2018). CB1R is widely expressed in the brain and regulates numerous physiological processes, including pain, neuroprotection, cognitive functions, motor activity, and feeding behavior (Howlett et al., 2002; Kano et al., 2009; Di Marzo et al., 2015). In contrast, CB2R is primarily expressed in immune cells where it mediates the anti-inflammatory and immunosuppressive effects of eCBs (Ashton and Glass, 2007). In the central nervous system (CNS), the behavioral and physiological effects of eCBs and exogenous cannabinoids can be largely ascribed to CB1R activation, although a contribution of CB2R has been suggested (Van Sickle et al., 2005; Sadanandan et al., 2020).

In the CNS, 2-AG and AEA act as retrograde messengers and activate presynaptic CB1Rs to decrease neurotransmitter release, thereby inducing short- and long-term eCB-dependent synaptic plasticity (Wilson and Nicoll, 2002). In addition to “on demand” (phasic) signaling, eCBs are also synthesized and constitutively released to mediate tonic control of synaptic transmission (Alger, 2014; Oubraim et al., 2021). Retrograde eCB signaling is tightly regulated by the enzymes that mediate their biosynthesis and inactivation. Postsynaptic 2-AG biosynthesis is catalyzed by diacylglycerol lipase alpha (DAGLα) whereas its degradation in presynaptic neurons and surrounding astrocytes is mediated by monoacylglycerol lipase (MAGL) (Murataeva et al., 2014). N-acyl phosphatidylethanolamine-specific phospholipase D (NAPE-PLD) and fatty acid amide hydrolase (FAAH) are the main biosynthetic and catabolic enzymes for AEA, respectively (Maccarrone, 2017).

The inherent lipophilicity of eCBs limits their transport across aqueous compartments, including the cytosol and synaptic cleft. We previously identified fatty acid-binding proteins (FABPs) as chaperones that facilitate the intracellular trafficking of eCBs to their respective catabolic enzymes and nuclear receptors (Kaczocha et al., 2009, 2012). Among the FABPs expressed in the brain (FABP3, FABP5, and FABP7) (Furuhashi and Hotamisligil, 2008), FABP5 has been shown to play a key role in gating eCB signaling and metabolism (Kaczocha et al., 2009; Haj-Dahmane et al., 2018). Indeed, we have shown that FABP5 is indispensable for retrograde 2-AG signaling at glutamatergic synapses in the dorsal raphe nucleus (DRn) (Haj-Dahmane et al., 2018). Although these recent findings point to a potential role of FABP5 as a facilitator of retrograde 2-AG signaling, it remains to be determined whether this function extends to AEA and 2-AG transport at synapses in other brain areas. Here, we profiled regional eCB levels and the expression of their metabolizing enzymes in WT and FABP5 KO mice and demonstrate a critical role for FABP5 in controlling retrograde AEA and 2-AG signaling at striatal GABA synapses.

## Materials and Methods

### Animals

All the procedures used in this study were approved by Stony Brook University (#1486041) and University at Buffalo (#RIA01023N) Animal Care and Use Committee in accordance with the National Institutes of Health Guide for the Care and Use of Laboratory Animals. Male and female wild-type (WT) C57BL/6 mice, as well as FABP5 KO mice on a C57BL/6 background (8–12 weeks), were group housed (3–4 per cage) with *ad libitum* access to food and water in temperature-controlled environment and 12-h light/dark cycle.

### Real-Time PCR

Ribonucleic acid (RNA) was extracted using the RNeasy Mini Kit (Qiagen) followed by complementary DNA (cDNA) synthesis using the SuperScript III First-Strand Synthesis System (Thermo Fisher). Real-time polymerase chain reaction (qPCR) was performed with PowerUp SYBR green (Thermo Fisher) on a StepOnePlus instrument (Applied Biosystems). Quantification was performed using the 2^−Δ*ΔCt*^ method with actin serving as the housekeeping gene. The following forward (F) and reverse (R) primers were used: FABP3: (F)CATCGAGAAGAACGGGGATA and (R)TCATCTGCTGTCACCTCGTC; FABP5: (F)TGGTCCAGCACCAGCAATG and (R)GACACACTCCACGATCATCTTC; FABP7: (F)CCAGCTGGGAGAAGAGTTTG and (R)TTTCTTTGCCATCCCACTTC; CB1R: (F)AAGTCGATCTTAGACGGCCTT and (R)TCCTAATTTGGATGCCATGTCTC; FAAH: (F)CCCTGCTCCAACTGGTACAG and (R)TCACAGTCAGTCAGATAGGAGG; MAGL: (F)CGGACTTCCAAGTTTTTGTCAGA and (R)GCAGCCACTAGGATGGAGATG; NAPE-PLD: (F)CTCCTGGACGACAACAAGGTTC and (R)GCAAGGTCAAAAGGACCAAAC; ABHD4: (F)TTCCCCTACGACCAACTGAC and (R)CGAAGAACAGCCAGTGGATT; ABHD6: (F)ACACAAGGACATGTGGCTCA and (R)ACTTGCCCCACTATGGACAG; DAGLα: (F)GTCCTGCCAGCTATCTTCCTC and (R)CGTGTGGGTTATAGACCAAGC; DAGLβ: (F)AGCGACGACTTGGTGTTCC and (R)GCTGAGCAAGACTCCACCG; and actin: (F)GACGGCCAGGTCATCACTAT and (R)CGGATGTCAACGTCACACTT.

### Lipid Quantification

Tissue eCB levels were quantified using mass spectrophotometry as previously described (Kaczocha et al., 2014). Brains were flash-frozen in liquid nitrogen followed by regional dissections on an ice block. The tissues were homogenized in 8 ml of 2:1:1 chloroform/ methanol/tris (50 mM, pH 8) containing deuterated standards, the phases were separated by centrifugation, and the chloroform phase was isolated and dried down under gentle argon stream. The samples were subsequently resuspended in 2:1 chloroform/methanol and injected into a Thermo TSQ Quantum Access Triple Quadrupole Mass Spectrometer (Thermo Fisher) and processed exactly as described previously (Kaczocha et al., 2014).

### Immunohistochemistry

Immunofluorescence was performed as described previously (Peng et al., 2017). Briefly, striatal sections (30 μm) were incubated with the following primary antisera: goat anti-FABP5 (R&D Systems Inc, #AF1476), mouse anti-NeuN (Millipore, MAB377), and mouse anti-s100β (Sigma, #S2532). Secondary antibodies used were Alexa Fluor 594 donkey anti-goat (Jackson ImmunoResearch Labs, #711-585-152) and Alexa Fluor 488 donkey anti-mouse IgG (H+L) antibody (Jackson ImmunoResearch Labs). Fluorescent images were acquired on a Zeiss Axioplan 2 epifluorescent microscope. Images were obtained using Zeiss AxioCam HRm monochrome digital camera and AxioVision Rel. 4.6 microscope software. Images were only adjusted for brightness and contrast.

### Brain Slice Preparation

Mice were deeply anesthetized with isoflurane and decapitated. Using a vibratome (Lancer series 1,000; Ted Pella, Reading, CA), coronal brain slices of 350 μm containing striatum were cut in ice-cold chlorine-based ACSF (110 mM choline-Cl, 2.5 mM KCl, 0.5 mM CaCl_2_, 7 mM MgSO_4_, 1.25 mM NaH_2_PO_4_, 26.2 mM NaHCO_3_, 11.6 mM sodium L-ascorbate, 3.1 mM sodium pyruvate, and 25 mM glucose, and equilibrated with 95% O_2_ and 5% CO_2_). Slices were first transferred to a chamber with the same cutting solution at 35°C for 15 min and then to a regular ACSF (119 mM NaCl, 2.5 mM CaCl_2_, 1.3 mM MgSO_4_, 1 mM NaH_2_PO_4_, 26.2 mM NaHCO_3_, and 11 mM glucose continuously bubbled with a mixture of 95% O_2_ and 5% CO_2_) for additional 45 min at 35°C. Slices were allowed to recover at room temperature (≥ 1 h), before being transferred to a recording chamber, continuously perfused with standard ACSF saturated with 95% O_2_ and 5% CO_2_, and warmed at 30°C.

### Whole-Cell Recordings

Whole-cell recordings were obtained from striatal medium spiny neurons (MSNs) and were visualized using an upright microscope (BX 51 WI; Olympus, Tokyo, Japan). Glass pipette electrodes with 3–5 MΩ resistance filled with an internal solution with the following composition: 110 mM cesium gluconate, 10 mM CsCl, 10 mM Na_2_-phosphocreatine, 10 mM HEPES, 1 mM MgCl_2_, 1 mM EGTA, 2 mM Na_2_-ATP, 0.25 mM Na-GTP, and 5 mM QX-314 chloride, pH 7.3 (adjusted with CsOH; osmolarity, 280–290 mOsmol/l), were used. All recordings were performed in the presence of NMDA and AMPA receptors antagonist D-AP5 (50 μM) and DNQX (10 μM).

Inhibitory postsynaptic currents (IPSCs) were evoked with a single square-pulse with a stimulation intensity of 5–20 V and duration of 100–200 μs, delivered at 0.1 Hz, and voltage-clamped at −80 mV. To induce depolarization-induced suppression of inhibition (DSI), IPSCs at 3-s intervals were evoked before (4 IPSCs) and after (20 IPSCs) 5-s depolarization from −80 to 0 mV. All recorded currents were amplified with an Axoclamp 2B amplifier (Molecular Devices, Sunnyvale, CA), filtered at 3 kHz, digitized at 20 kHz with Digidata 1200 (Molecular Devices), and acquired using the pClamp 9 software (Molecular Devices).

### Electrophysiology Data Analysis

The amplitude of evoked IPSCs (eIPSC) was determined by measuring the average current during a 2-ms period at the peak of each eIPSC and subtracted from the average baseline current determined during a 5-ms window taken before the stimulus artifact. All eIPSC amplitudes were normalized to the mean baseline amplitude recorded for at least 10 min before drug application. The magnitude of DSI was measured using the mean amplitude of three eIPSCs immediately after membrane depolarization, relative to the mean of four before depolarization.

### Statistical Analysis

Real-time PCR and lipidomic results were analyzed using unpaired *t*-tests. Electrophysiology data were analyzed using paired *t*-tests within group comparisons and unpaired *t*-tests between groups with a significance threshold set at *p* < 0.05. Statistical analyses were performed using GraphPad Prism (version 9.3.1) and Origin software (version 9.0).

## Results

### Comparative FABP5 Expression Across Brain Regions in Male and Female Mice

**Figure 1:**
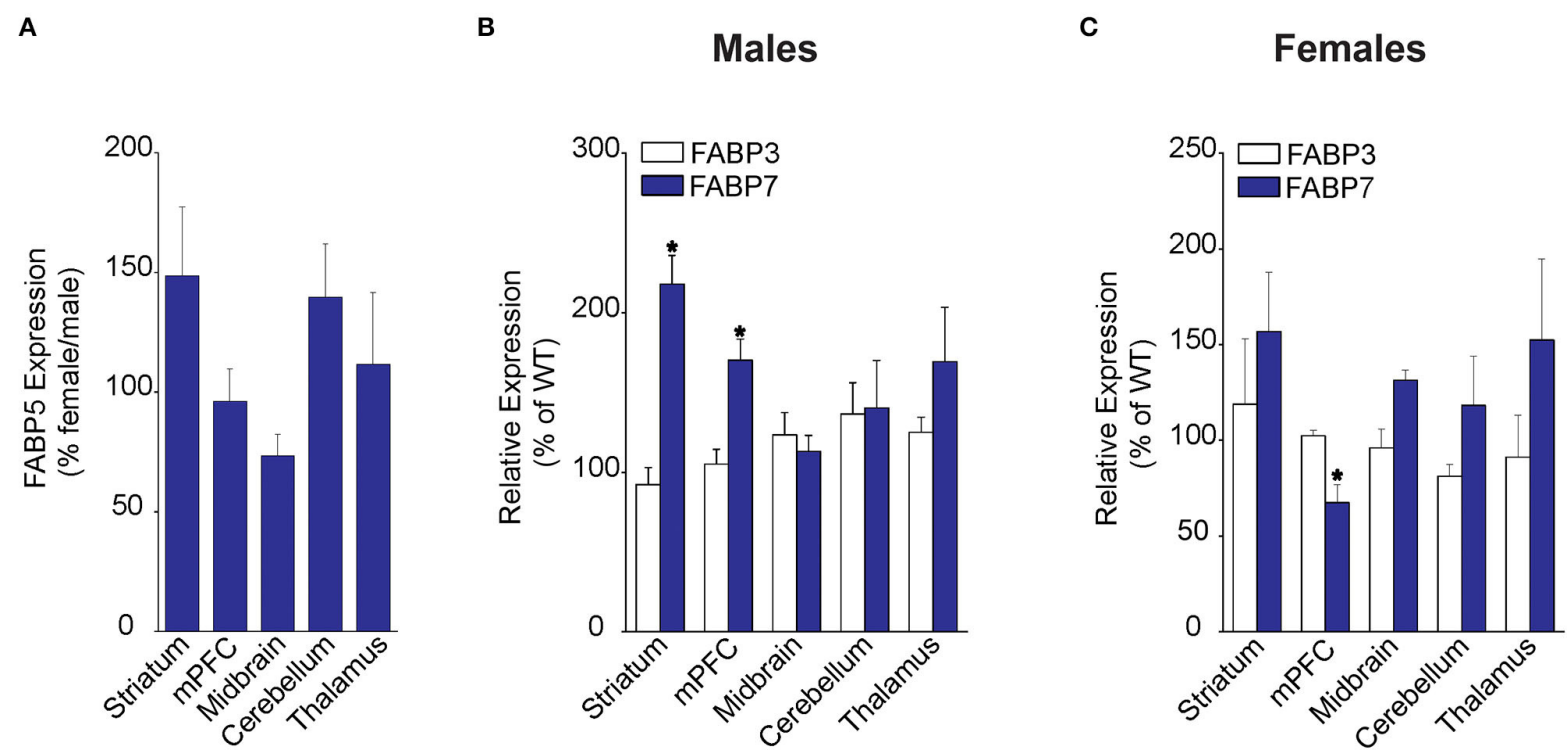
Regional FABP expression in male and female WT and FABP5 KO mouse brains. **(A)** Comparative FABP5 expression in WT male and female mice in various brain areas was assessed by qPCR (*n* = 5). Data are presented as percent expression in female/male mice. **(B,C)** Relative expression of FABP3 and FABP7 in male and female FABP5 KO mice compared to WT controls (*n* = 5). *, *p* < 0.05; KO vs. WT.

We first examined FABP5 expression in WT male and female mice across several brain regions, including striatum, medial prefrontal cortex (mPFC), midbrain, cerebellum, and the thalamus. Our results did not reveal the sex differences in relative FABP5 expression in the regions examined (Figure 1A). Next, we examined whether FABP5 deletion alters the expression of FABP3 and FABP7 and found comparable expression of FABP3 in male and female WT and FABP5 KO mice (Figures 1B,C). In contrast, an upregulation of FABP7 was observed in the striatum and mPFC of male FABP5 KO mice and downregulation in the mPFC of female FABP5 KO mice (Figures 1B,C). Collectively, our findings indicate comparable FABP5 expression between the sexes and reveal region-specific, as well as sexually dimorphic, compensatory changes in FABP7 levels.

### FABP5 Deletion Elevates AEA and 2-AG Levels in Distinct Brain Regions

**Figure 2:**
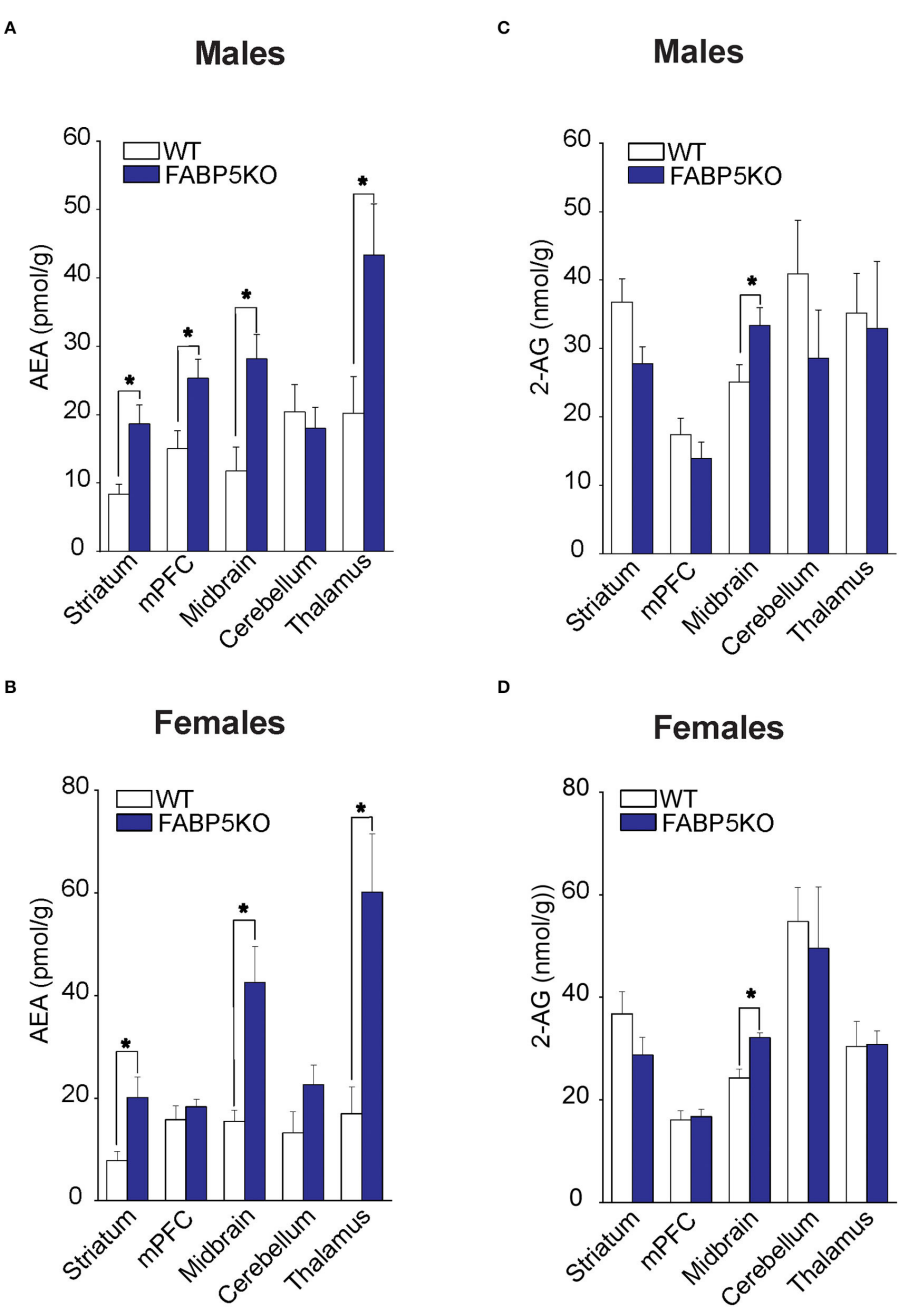
2-AG and AEA levels in brain regions of WT vs. FABP5 KO mice. Levels of AEA **(A,B)** and 2-AG **(C,D)** in the striatum, mPFC, midbrain, cerebellum, and thalamus of male and female WT and FABP5 KO mice (*n* = 5). *, *p* < 0.05; KO vs. WT.

Fatty acid-binding protein 5 inhibition globally increases AEA levels in the mouse brain (Kaczocha et al., 2014). To explore whether elevations in AEA exhibit regional heterogeneity, we quantified AEA levels in the same brain areas as mentioned above. Compared to WT mice, elevated AEA levels were observed in the striatum, midbrain, and thalamus of male and female FABP5 KO mice (Figures 2A,B). Interestingly, AEA levels were elevated in the mPFC of male mice, but not in female FABP5 KO mice (Figures 2A,B). Previous work revealed increased 2-AG levels in the DRn of FABP5 KO mice (Haj-Dahmane et al., 2018). Similarly, we found that 2-AG levels were elevated in the midbrain of male and female FABP5 KO mice whereas no differences were observed in the other regions profiled (Figures 2C,D).

### FABP5 Deletion Does Not Alter the Expression of Biosynthetic Enzymes for 2-AG and AEA

**Figure 3:**
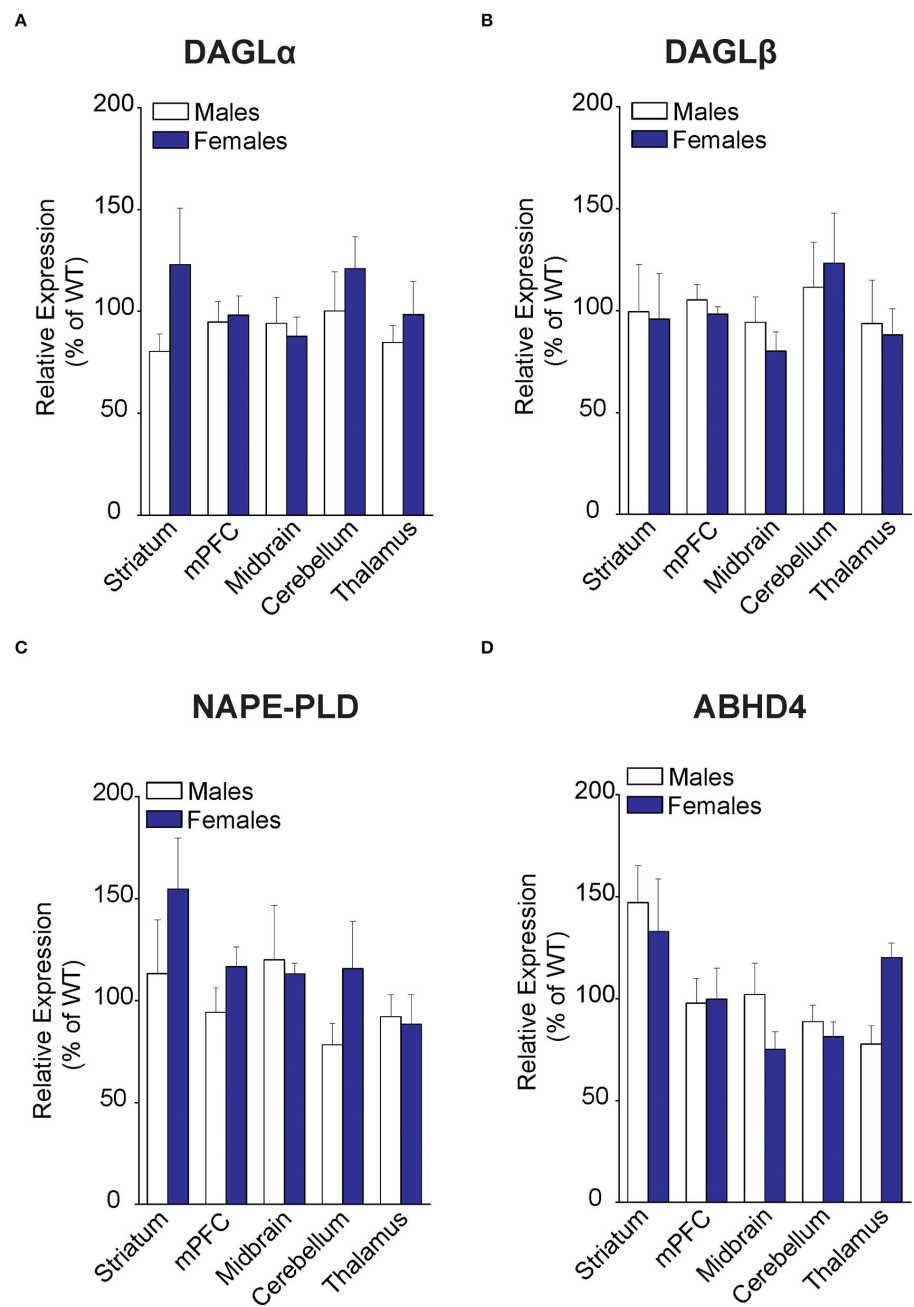
Expression of 2-AG and AEA biosynthetic enzymes in brain regions of WT and FABP5 KO mice. **(A–D)** Relative expression of DAGLα, DAGLβ, NAPE-PLD, and ABHD4 in male and female FABP5 KO mice compared to WT controls (*n* = 5). Note that FABP5 deletion did not significantly alter the expression of these biosynthetic enzymes.

To determine whether the changes in eCB levels in FABP5 KO mice can be attributed to differential expression of their metabolizing enzymes, we first profiled eCB biosynthetic enzymes in the same regions as above. Comparable expression of DAGLα and DAGLβ was observed in the striatum, mPFC, midbrain, cerebellum, and thalamus of WT and FABP5 KO mice of both sexes (Figures 3A,B). Similarly, there was no change in the expression of NAPE-PLD and alpha-beta hydrolase domain containing 4 (ABHD4), which mediate the canonical and alternative AEA biosynthetic pathways, respectively (Figures 3C,D).

### FABP5 Deletion Results in Sex- and Region-Specific Alterations in the Expression of CB1R and 2-AG Catabolic Enzymes

**Figure 4:**
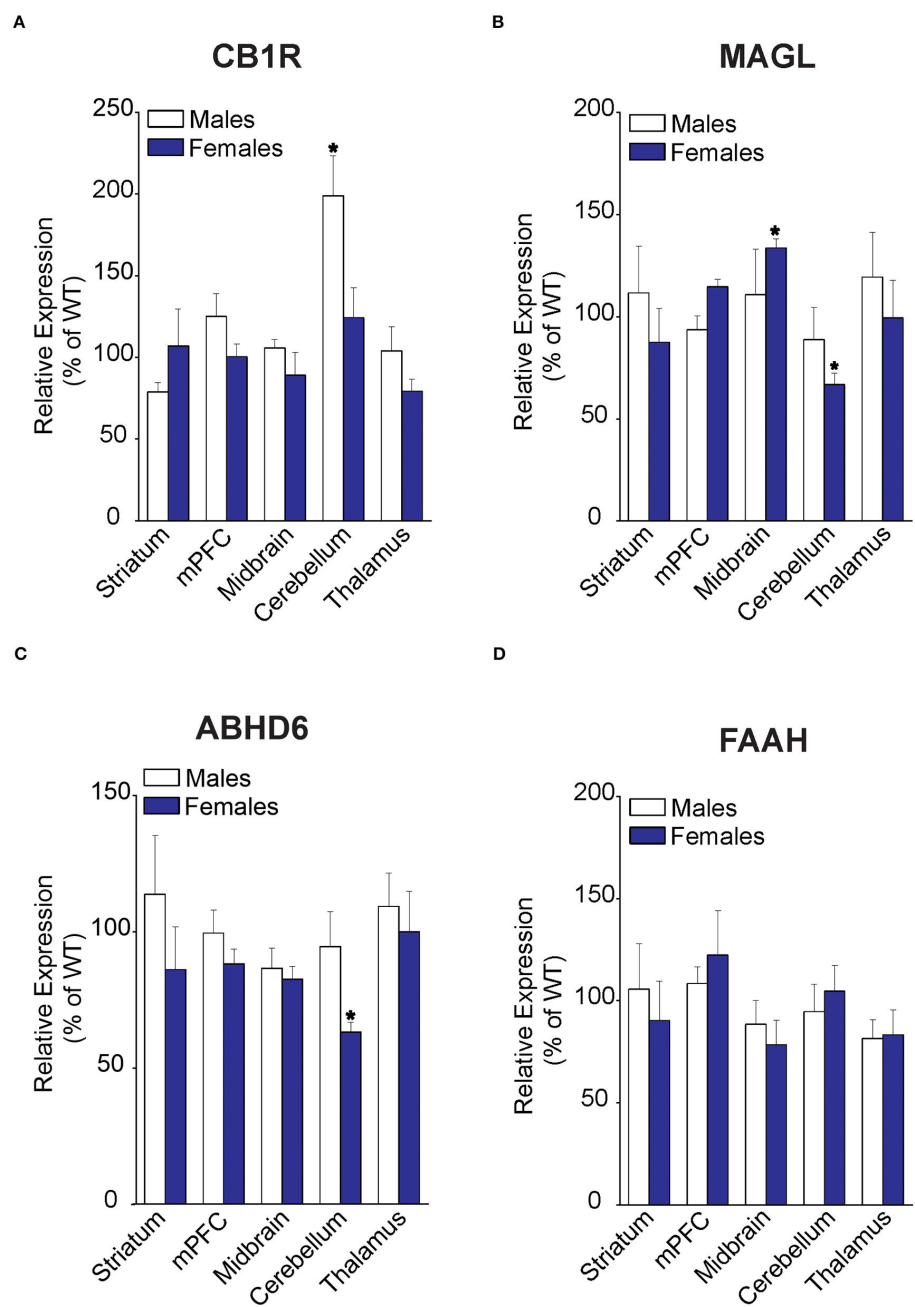
Relative expression of 2-AG and AEA catabolic enzymes in brain regions of WT and FABP5 KO mice. **(A–D)** Relative expression of CB1R, MAGL, ABHD6, and FAAH in male and female FABP5 KO mice compared to WT controls (*n* = 5). *, *p* < 0.05; KO vs. WT.

Examination of CB1R expression revealed comparable levels across the brain regions, with the sole exception of CB1R upregulation in the cerebellum of male FABP5 KO mice (Figure 4A). Next, an analysis of 2-AG catabolic enzymes demonstrated MAGL upregulation in the midbrain and downregulation of both MAGL and ABHD6, a minor brain 2-AG hydrolase, in the cerebellum of female FABP5 KO mice (Figures 4B,C). In contrast, the expression of the AEA catabolic enzyme FAAH was not altered in any of the regions examined (Figure 4D).

### Effect of FABP5 Deletion on Tonic and Phasic eCB Signaling

**Figure 5:**
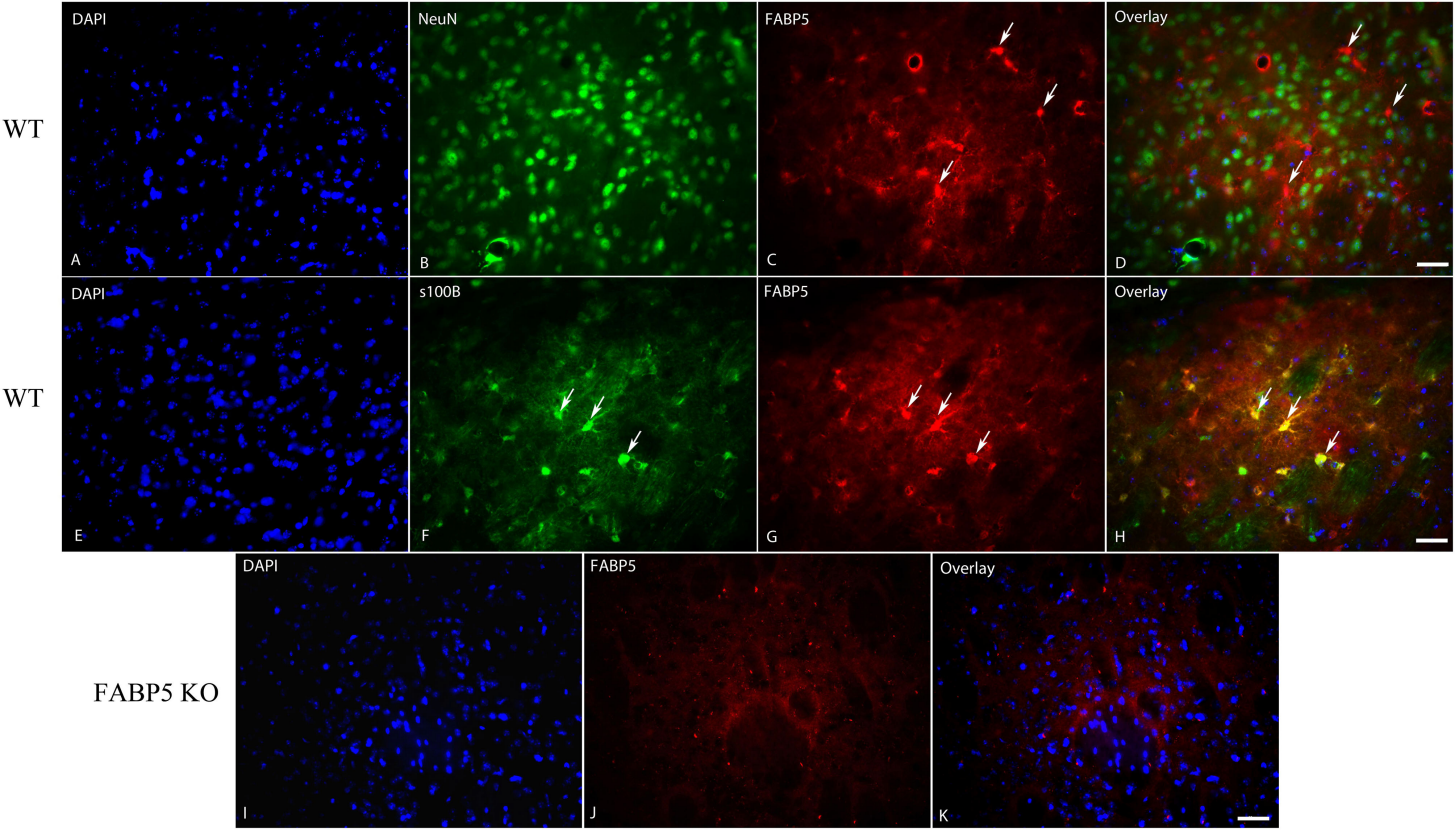
Cellular distribution of FABP5 in the dorsal striatum. Immunolocalization of FABP5 in the striatum of WT mice. Staining for DAPI **(A)**, the neuronal marker NeuN **(B)**, FABP5 **(C)**, and overlay **(D)**. Note the minimal colocalization of FABP5 in NeuN labeled neurons (arrows). Staining for DAPI **(E)**, the astrocyte marker S100β **(F)**, FABP5 **(G)**, and overlay **(H)**. Note that there is robust colocalization between FABP5 and S100β (arrows), indicating that FABP5 is expressed in astrocytes. Immunostaining was conducted on striatal sections from three WT mice and representative images are shown. **(I–K)** The lack of FABP5 immunoreactivity in the striatum of FABP5 KO mice. Scale bar = 20 μm.

We previously demonstrated that FABP5 controls retrograde 2-AG signaling at glutamate synapses in the DRn (Haj-Dahmane et al., 2018). However, it is currently not known whether FABP5 regulates retrograde eCB signaling in other brain areas, and notably at inhibitory synapses. The finding that FABP5 deletion elevated striatal AEA levels coupled with previous reports demonstrating retrograde AEA, as well as 2-AG signaling in this region (Adermark and Lovinger, 2007; Hashimotodani et al., 2007, 2008, 2013; Adermark et al., 2009), prompted us to examine FABP5 distribution and its influence upon eCB signaling within this area. Robust FABP5 expression was observed in this region and was largely restricted to astrocytes as indicated by colocalization with the astrocyte marker s100β (Figure 5). In contrast, FABP5 rarely colocalized with the neuronal marker NeuN (Figure 5).

**Figure 6:**
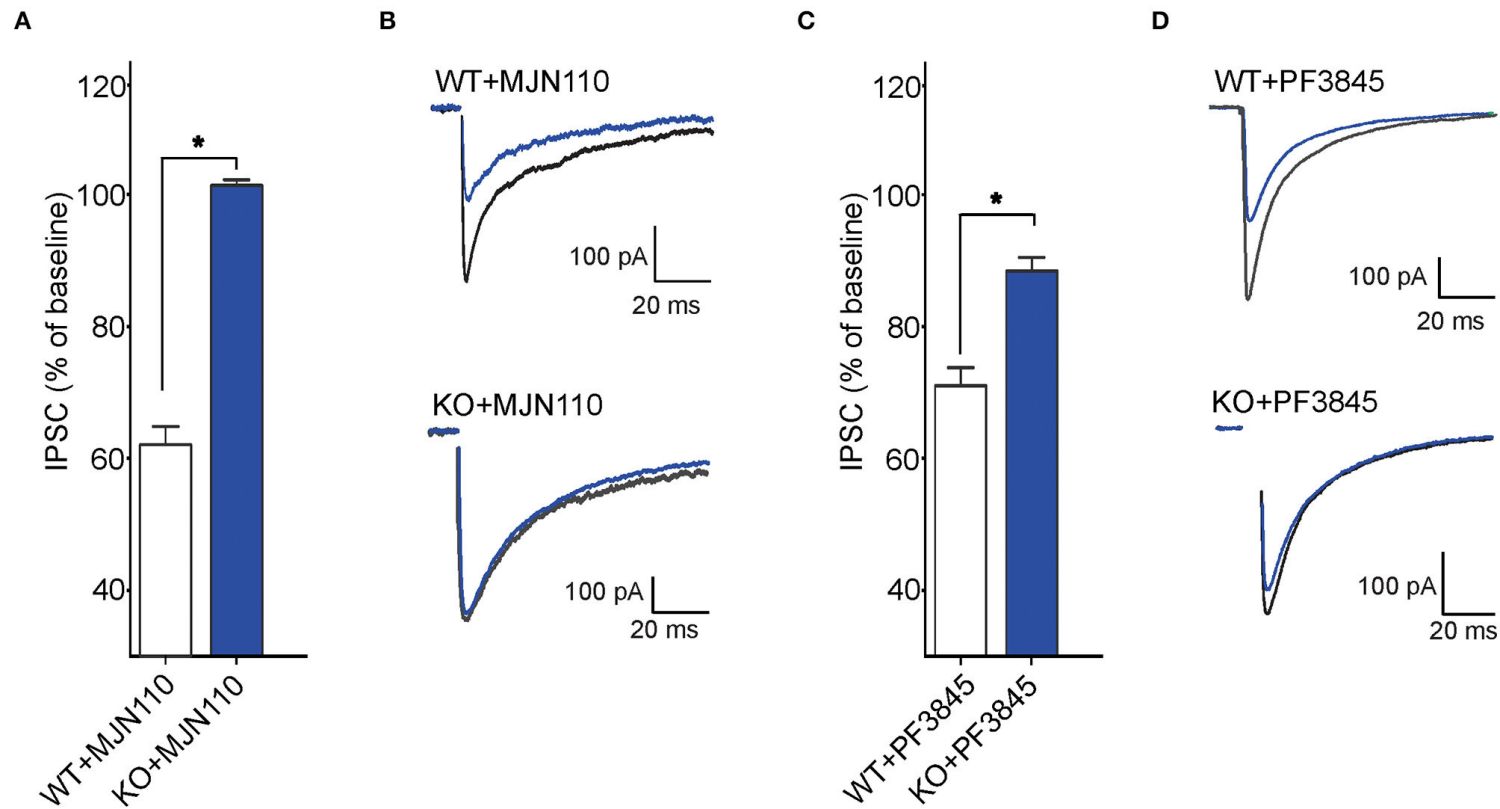
FABP5 deletion impairs tonic 2-AG and AEA signaling at striatal GABA synapses. **(A)** The average depression of eIPSC induced by the MAGL inhibitor MJN110 (10 μM) in WT and FABP5 KO mice. Average reduction in the amplitude of eIPSCs to baseline, in WT (WT+MJN110: 62.01 ± 1.10%, *n* = 5 cells from 5 mice, t4 = 17.83, *p* < 0.05, vs. baseline) and FABP5 KO (KO+MJN110: 101.36 ± 0.32 %, *n* = 5 cells from 5 mice, t4 = −0.52, *p* > 0.05, vs. baseline) slices during bath application of MAGL inhibitor. **(B)** Sample eIPSC traces taken before and during MJN110 application in WT (top) and FABP5 KO mice (bottom). Note that FABP5 deletion prevents the depression of eIPSCs induced by MJN110. **(C)** Average reduction of eIPSC induced by the FAAH inhibitor PF3845 (3 μM) in WT (WT+PF3845: 71.14 ± 1.16%, n = 5 cells from five mice, t4 = 18.49, *p* < 0.05 vs. baseline) and FABP5 KO (KO+PF3845: 88.52 ± 0.74 %, n = 5 cells from 5 mice, t4 = 5.10, *p* < 0.05, vs. baseline) slices. **(D)** Representative eIPSC traces before and during PF3845 application in WT and FABP5 KO mice. *, *p* < 0.05.

Next, we examined tonic retrograde eCB signaling by assessing the depression of GABA synapses induced by MAGL or FAAH inhibition in WT and FABP5 KO mice. Pharmacological inhibition of MAGL using MJN110 (Figures 6A,B) or FAAH using PF3845 (Figures 6C,D) induced significant depression of evoked inhibitory postsynaptic currents (eIPSCs) in WT mice, indicating that both 2-AG and AEA mediate tonic control of striatal GABA synapses. Remarkably, FABP5 deletion blunted MJN110-induced depression of eIPSCs (Figures 6A,B) and markedly reduced the effect of PF3845 on eIPSCs amplitude (Figures 6C,D). These results indicate that FABP5 deletion impairs tonic AEA and 2-AG signaling at striatal GABA synapses.

**Figure 7:**
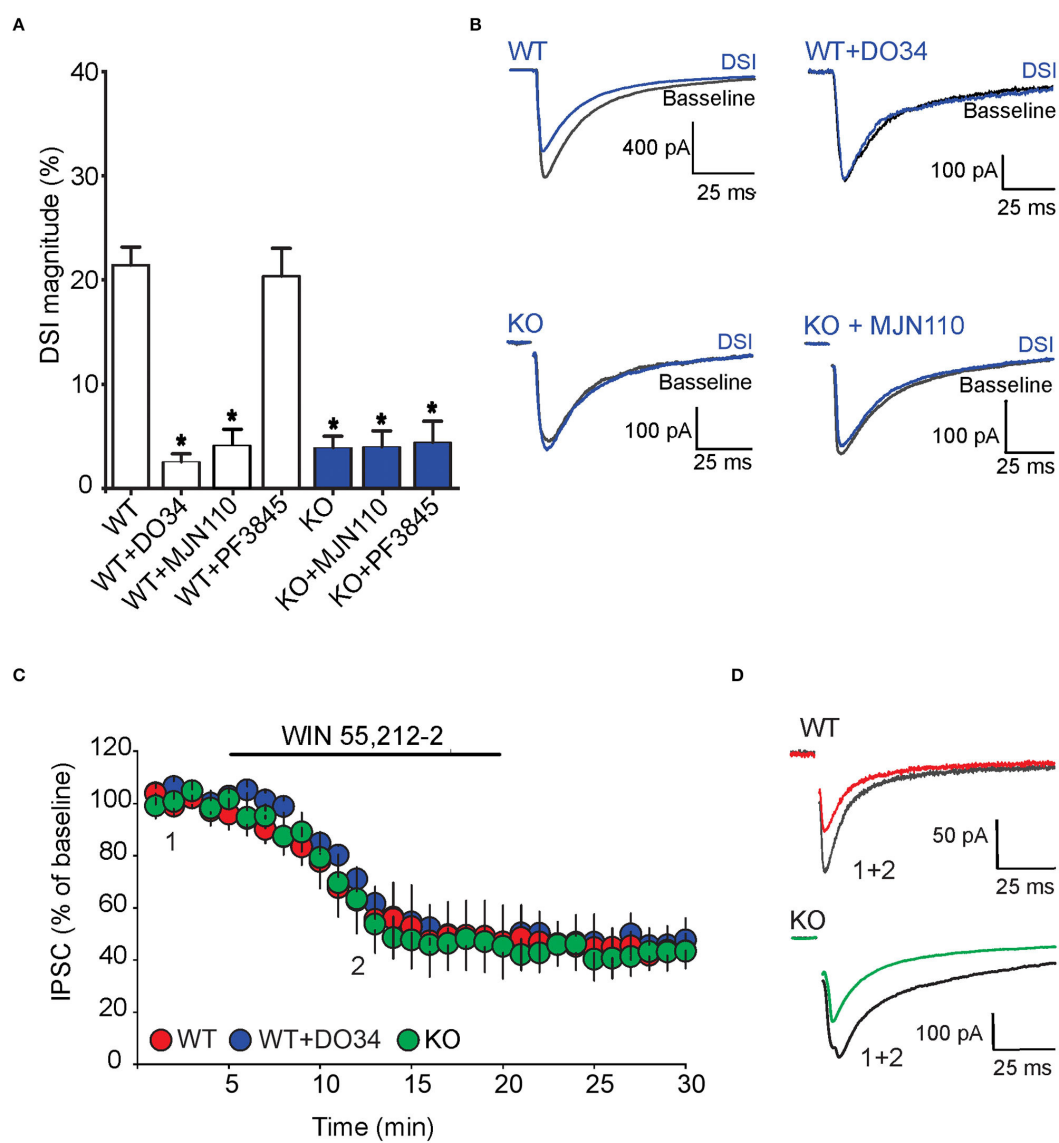
FABP5 deletion blunts 2-AG mediated DSI without altering CB1R function in striatal MSNs. **(A)** DSI magnitude obtained in striatal slices from WT (white bars) and FABP5 KO mice (blue bars), incubated with vehicle (WT: 21.43 ± 1.70%, *n* = 12 cells from 10 mice; KO: 3.90 ± 1.08%, *n* = 12 cells from 10 mice), DO34 (1 μM) (WT + DO34: 2.53 ± 0.75%, *n* = 8 cells from 6 mice, *p* < 0.05 vs. WT), MJN110 (10 μm) (WT+MJN110: 4.14 ± 1.45%, *n* = 7 cells from 6 mice, *p* < 0.05 vs. WT), or PF3845 (3 μM) (WT+PF3845: 20.35 ± 2.66%, *n* = 7 cells from 6 mice, *p* > 0.05 vs. WT, *p < 0.05 vs WT). **(B)** Sample eIPSC traces before and during DSI in WT, WT + D034, FABP5 KO, FABP5 KO + MJN110. **(C)** The depression of eIPSCs induced by the CB1R agonist WIN55,212-2 (10 μM) in striatal slices from WT (red) (WT: 47.81 ± 11.80% of baseline, *n* = 9 cells from 5 mice, t_8_ = 4.28, *p* < 0.01, vs. baseline), WT + DO34 (blue) (WT+DO34: 49.75 ± 6.06% baseline, *n* = 8 cells from 5 mice, t_7_ = 9.84, *p* < 0.001, vs. baseline), or FABP5 KO mice (green) (FABP5 KO: 45.18 ± 10.61% baseline, *n* = 8 cells from 5 mice, t_7_ = 5.11, *p* < 0.01, vs. baseline). Note that FABP5 deletion or inhibition of DAGL did not alter CB1R function. **(D)** Sample eIPSC traces taken before (1) and during (2) WIN55,212-2 application in WT and FABP5 KO striatal slices.

We also examined the impact of FABP5 deletion on phasic eCB signaling as measured by depolarization-induced suppression of inhibition (DSI) (Narushima et al., 2006, 2007; Uchigashima et al., 2007). As expected, we observed DSI in GABA striatal MSNs (Figures 7A,B). Examination of which eCB mediates DSI revealed that inhibition of DAGLα with DO34 (1 μM) significantly reduced the magnitude of DSI (Figure 7). Furthermore, inhibition of MAGL with MJN110, which reduced the baseline amplitude of GABA eIPSCs (Figure 6), largely occluded DSI (Figures 7A,B), confirming that it is mediated by 2-AG. In contrast, inhibition of FAAH using PF3845 did not alter the magnitude of the DSI (Figures 7A,B). Importantly, genetic deletion of FABP5 abolished DSI in MSNs (Figures 7A,B), which was not further altered by MJN110 or PF3845. Collectively, these results reveal that FABP5 is essential for both tonic and phasic eCB signaling in the striatum.

To test whether the blockade of tonic and phasic eCB signaling observed in FABP5 KO mice can be attributed to altered CB1R function, we measured the inhibition of GABA eIPSCs induced by the exogenous CB1R agonist WIN55,212-2 (10 μM) in WT and FABP5 KO mice. The magnitude of CB1R-induced inhibition of eIPSCs was comparable in WT and FABP5 KO mice, thereby indicating no change in CB1R function between the genotypes (Figures 7C,D). Similarly, inhibition of DAGLα with DO34 did not affect the function of CB1R (Figures 7C,D). Taken together, these results indicate that the blockade of retrograde eCB signaling in FABP5 KO mice cannot be attributed to impaired CB1R function, but rather to impaired eCB trafficking.

## Discussion

The eCBs are essential messengers that fine-tune synaptic transmission and plasticity in the CNS. The lipophilic nature of eCBs necessitates a mechanism(s) that enables their translocation across the synaptic cleft from their site of synthesis to CB1Rs. Several mechanisms have been proposed to account for synaptic eCB transport, including FABP5 and extracellular vesicles (Kaczocha and Haj-Dahmane, 2021). We have previously shown that FABP5 is necessary for retrograde 2-AG signaling at glutamate synapses of DRn neurons (Haj-Dahmane et al., 2018). Here, we extend these findings and show that FABP5 is also indispensable for tonic and phasic 2-AG signaling at striatal GABA synapses, and for the first time reveal that FABP5 governs tonic AEA signaling.

Fatty acid-binding protein 5 has an established role in mediating intracellular AEA transport, thereby gating its subsequent metabolism by FAAH (Kaczocha et al., 2009). Accordingly, FABP5 inhibition elevates AEA levels in the whole brain (Kaczocha et al., 2014; Yu et al., 2014). Consistent with this function, our results revealed that AEA levels were elevated in the midbrain, striatum, and thalamus of both male and female KOs. Interestingly, AEA levels were increased in the mPFC of male mice, but not in female FABP5 KO mice. Since FAAH, NAPE-PLD, and ABHD4 expression is comparable between the sexes and genotypes, these sex-specific differences in AEA levels are unlikely to arise from altered expression of its biosynthetic or catabolic enzymes. We also observed that deletion of FABP5 resulted in a compensatory increase in FABP7 expression within the mPFC of male mice and a corresponding decrease in female FABP5 KO mice, suggesting compensatory adaptations in response to the loss of FABP5. Moreover, although upregulation of FABP7 was observed in the striatum of male FABP5 KO mice, we observed no differences in 2-AG levels between male and female FABP5 KO mice, arguing against a role for this protein in 2-AG metabolism. However, we cannot rule out the possibility that the observed changes in mRNA expression may not translate to altered protein levels. One interesting finding of our study is that cerebellar AEA levels were unaltered in FABP5 KO mice of both sexes, consistent with a previous study reporting low levels of FABP5 expression in this region (Owada et al., 1996).

The results from previous studies indicate that retrograde AEA and 2-AG gate synaptic transmission in the striatum (Adermark and Lovinger, 2007; Hashimotodani et al., 2007, 2008, 2013; Adermark et al., 2009). As expected, we found that striatal GABA synapses are tonically modulated by AEA and 2-AG in WT mice as revealed by FAAH and MAGL inhibitor-induced depression of eIPSC amplitude. Genetic deletion of FABP5, which elevated striatal AEA levels, profoundly reduced AEA-mediated tonic signaling. This finding may reflect impaired intracellular AEA trafficking to FAAH for inactivation and synaptic transport to CB1R. In addition, whereas FABP5 deletion did not alter striatal 2-AG levels, it completely blocked tonic 2-AG mediated control of GABA synapses. This indicates that synaptic changes in 2-AG transport and signaling following FABP5 deletion may not be consistently reflected when quantifying bulk tissue 2-AG levels.

Employing selective DAGL, MAGL, and FAAH inhibitors, we further showed that striatal DSI is mainly attributable to retrograde 2-AG signaling. Genetic deletion of FABP5 blocked 2-AG-mediated DSI, an effect that was not a consequence of a reduction in 2-AG levels, altered expression of its biosynthetic or catabolic enzymes, or downregulation of CB1R. These findings are consistent with our previous results showing that pharmacological and genetic FABP5 inhibition abolishes 2-AG mediated tonic and phasic signaling at glutamate synapses in the DRn (Haj-Dahmane et al., 2018). Taken together, these studies establish FABP5 as a key regulator of synaptic eCB trafficking at both excitatory and inhibitory synapses in multiple brain areas.

Finally, we observed that FABP5 is mainly expressed in striatal astrocytes, which is distinct from other brain areas where neuronal FABP5 expression was reported (Owada et al., 1996; Haj-Dahmane et al., 2018). However, it is possible that FABP5 is expressed in presynaptic terminals, and additional high-resolution imaging studies will be required to test this possibility. Importantly, we previously demonstrated that primary astrocytes in culture secrete FABP5 whereas neurons do not (Haj-Dahmane et al., 2018), raising the intriguing possibility that FABP5 released from astrocytes may mediate synaptic AEA and 2-AG signaling in the striatum. Future studies addressing the contributions of astrocytic and neuronal FABP5 in controlling retrograde eCB signaling are required to test this notion.

## Data Availability Statement

The original contributions presented in the study are included in the article/supplementary materials, further inquiries can be directed to the corresponding authors.

## Ethics Statement

The animal study was reviewed and approved by Animal Care and Use Committee in accordance with the National Institutes of Health Guide for the Care and Use of Laboratory Animals.

## Author Contributions

MF conducted biochemical and histological experiments. SO conducted the *in vitro* slice electrophysiology studies. MY and SG performed qPCR experiments. MF, SO, MK, and SH-D designed the study, analyzed the data, and wrote the manuscript. All authors contributed to the article and approved the submitted version.

## Funding

This study was supported by the NIH grants: MH122461 (to SH-D and MK) and DA035949 (to MK).

## Conflict of Interest

The authors declare that the research was conducted in the absence of any commercial or financial relationships that could be construed as a potential conflict of interest.

## Publisher's Note

All claims expressed in this article are solely those of the authors and do not necessarily represent those of their affiliated organizations, or those of the publisher, the editors and the reviewers. Any product that may be evaluated in this article, or claim that may be made by its manufacturer, is not guaranteed or endorsed by the publisher.
